# Objections to the Vegetable Origin of Porrigo Decalvans

**Published:** 1846-04

**Authors:** 


*Objections to the Vegetable Origin of Porrigo Decalvans.*—Mr. Horne writes a letter in the Lancet, containing what appears to us very unanswerable objections to some observations of M. Gruby, quoted in our last number, purporting to prove the vegetable origin of Porrigo Decalvans. Mr. Horne remarks :—

"The first objection I have to make is, that instead of the bald surface being covered with dry scales, or a white bran-like powder, as M. Gruby supposes, this condition never exists in the disease under consideration, the external appearances of porrigo decalvans presenting a bald, smooth, and remarkably shining indentated surface.

"If the disease produces no powdery secretion, we have none to examine ; therefore I pass to the second objection,—'The hairs usually break off at the point where they are invested with the vegetable covering.'

"In porrigo decalvans the hair falls out, bringing the bulb with it, through absorption of the adipose structure, in which the bulbs are included, and from which the hair draws its nourishment ; the absorption of this matter produces the remarkable indentation or flattening in each bald patch, and constitutes the pathological nature of the disease.

"Now, in the common ringworm of the scalp, the porrigo scutulata of Willan, to whom we are indebted for the term decalvans, the hair does fall or break off, and is covered with an incrustation.

"Third objection: 'the vegetable nature of porrigo decalvans is a fact that speaks for the contagiousness of the disease.' Now, porrigo decalvans is not contagious, and this the professor may take for granted, as my opinion is not grounded on the result of a few stray cases, but from facts observed daily in this particular disease for some years back. The conclusion that I have come to is, that M. Gruby, and the promulgators of his hypothesis, have confounded one disease with another. I do not say with the porrigo scutulata only, for there are other diseases of the scalp, with which those not practically acquainted with them, may very easily confound it."—*Lancet*, Feb. 10, 1844.

Mr. Horne has M. Gruby completely on the hip.—*Medico-Chirurgical Review.*

